# Pachydermoperiostosis (Touraine–Solente–Gole syndrome): A case report and literature review

**DOI:** 10.1097/MD.0000000000049450

**Published:** 2026-06-19

**Authors:** Xiaohan Hu, Min Li, Zhou Zhou

**Affiliations:** a Department of Dermatology, Chinese People’s Liberation Army Western Theater Command General Hospital, Chengdu, Sichuan, China.

**Keywords:** case report, digital clubbing, hypertrophic osteoarthropathy, pachydermoperiostosis

## Abstract

**Rationale:**

Pachydermoperiostosis (PDP) is a rare genetic disorder characterized by the triad of pachyderma, digital clubbing, and periostosis. We report the case of a 17-year-old male who presented with classic clinical features over 3 years, including progressive skin thickening on the forehead, joint pain and swelling, hyperhidrosis, and facial oiliness, leading to a clinical and radiological diagnosis of PDP.

**Patient concerns:**

The patient exhibited pronounced forehead folds, oily skin with acneiform lesions, digital clubbing, and severe bilateral knee swelling.

**Diagnoses:**

Laboratory investigations and most imaging studies were unremarkable. Crucially, lower leg radiographs identified cortical periosteal thickening. Genetic testing for 15-hydroxyprostaglandin dehydrogenase (HPGD) and Solute Carrier Organic Anion Transporter Family Member 2A1 (SLCO2A1) mutations was recommended but declined. A diagnosis of PDP was made based on the classic clinical triad.

**Interventions:**

The patient was treated with oral isotretinoin (20 mg once daily) and diclofenac (50 mg twice daily).

**Outcomes:**

Post-treatment, the patient reported improvement in facial oiliness and joint symptoms. During a five-year follow-up, the patient’s condition stabilized with no signs of regression.

**Lessons:**

Diagnosis is primarily clinical and radiological; genetic testing for HPGD/SLCO2A1 mutations can confirm the diagnosis but was declined in this case. Management relies on non-steroidal anti-inflammatory drugs for joint symptoms, with isotretinoin offering additional benefit for cutaneous manifestations. Long-term follow-up is advised.

## 1. Introduction

Pachydermoperiostosis (PDP), or Touraine-Solente-Gole syndrome, is a rare primary form of hypertrophic osteoarthropathy (HOA) characterized by the triad of pachyderma (skin thickening), digital clubbing, and periostosis.^[1]^ It is genetically heterogeneous, primarily involving mutations in the 15-hydroxyprostaglandin dehydrogenase (HPGD) or Solute Carrier Organic Anion Transporter Family Member 2A1 (SLCO2A1) genes, which disrupt prostaglandin E2 (PGE2) metabolism, leading to its systemic accumulation.^[2,3]^ Most cases manifest during adolescence and progress over a decade before stabilizing.^[4]^

## 2. Case presentation

### 2.1. Clinical presentation

A 17-year-old male presented with a 3-year history of gradually progressive skin thickening and wrinkling on the forehead. He concurrently reported intermittent pain and swelling of both knees, excessive sweating of the palms and soles, and increased facial oiliness. There was no family history of similar conditions, and he was the only child of non-consanguineous parents.

**Figure 1.**
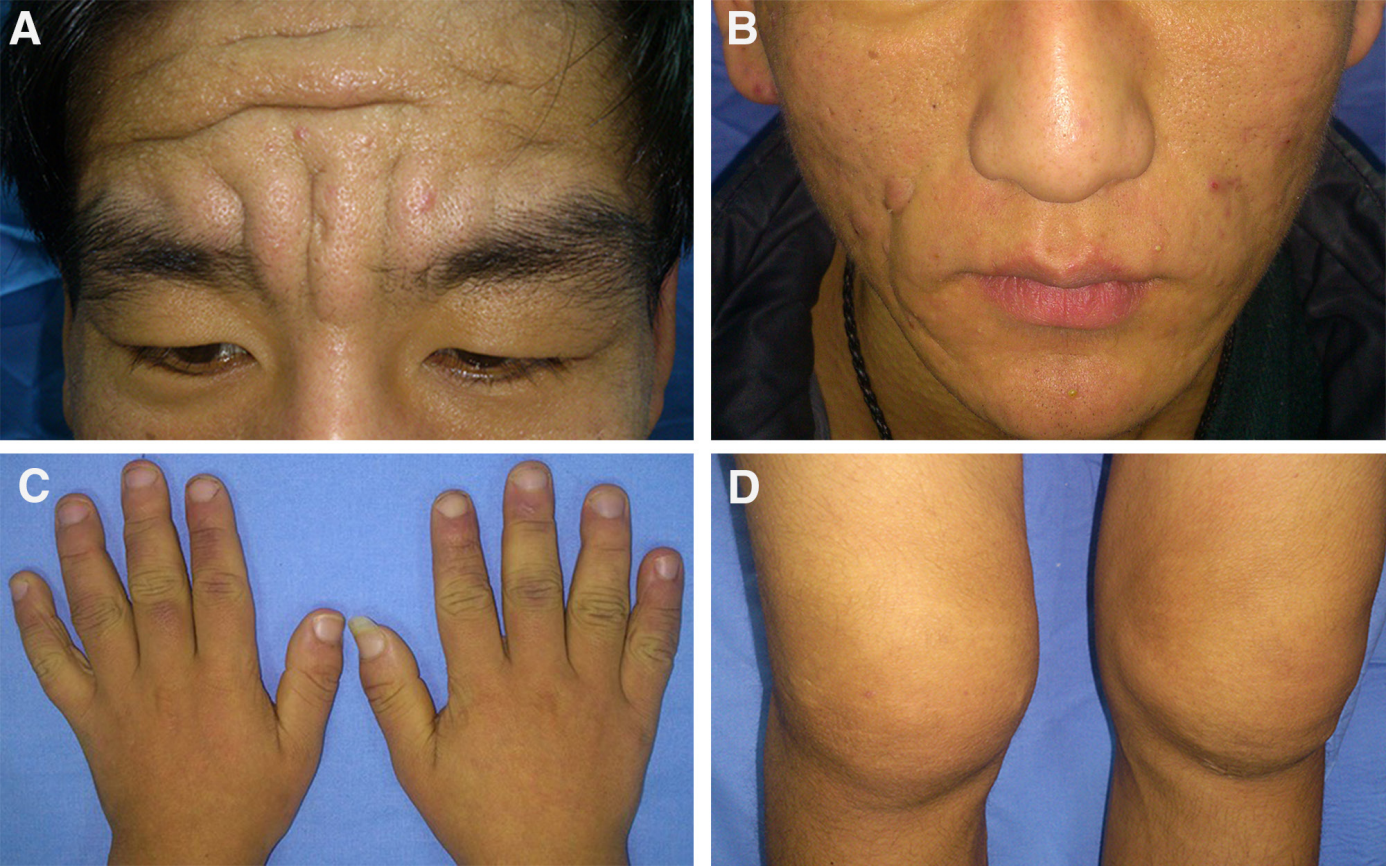
(A) Forehead skin hypertrophy; (B) perioral papules, pustules, and nodules; (C) hand enlargement with digital clubbing; (D) swollen knees.

Physical examination revealed a normocephalic and normally intelligent individual. Marked longitudinal and transverse folds were evident on the forehead and glabellar region (Fig. 1A). The facial skin was oily, with multiple perioral papules, pustules, and nodules (Fig. 1B). The scalp skin was thickened and loosely adherent to the underlying calvarium. Spade-like enlargement of the hands and feet with obvious digital clubbing was noted (Fig. 1C). Severe bilateral knee swelling was present (Fig. 1D).

### 2.2. Laboratory and imaging findings

Laboratory investigations, including serum growth hormone, thyroid function, syphilis serology, and rheumatoid factor, were within normal limits. Serum alkaline phosphatase (ALP) was 125 U/L. Bone mineral density (BMD) assessment of the lumbar spine and femoral neck showed values appropriate for his age and sex.

**Figure 2.**
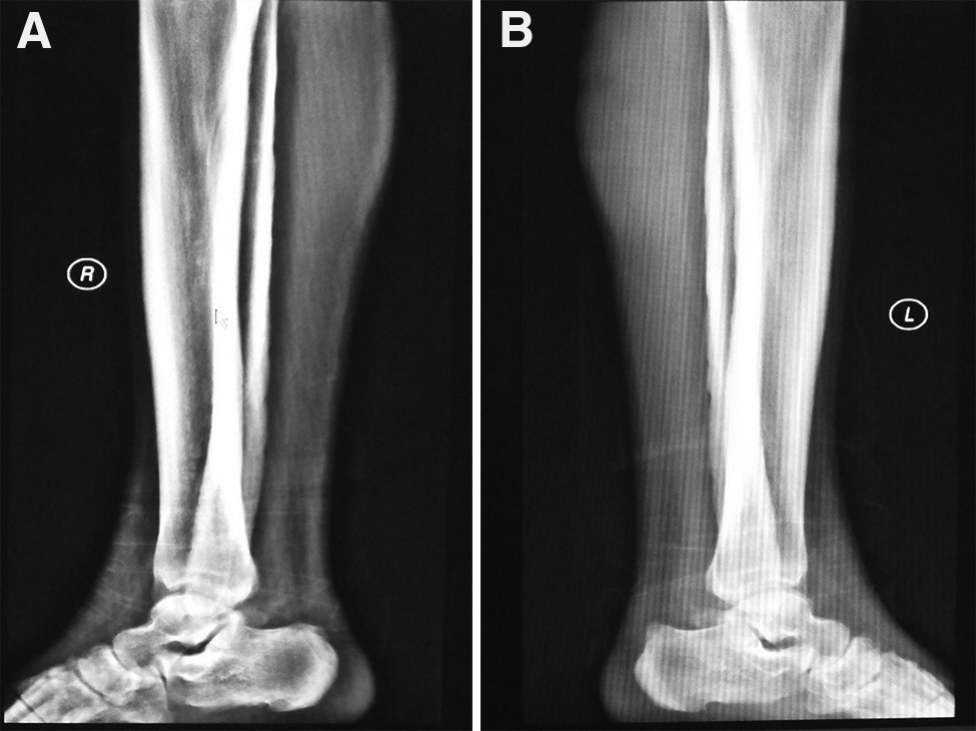
(A) Radiograph illustrating periostosis of the right lower leg; (B) radiograph depicting periostosis of the left lower leg.

Imaging studies yielded largely unremarkable results: echocardiography, abdominal ultrasonography, magnetic resonance imaging of the pituitary fossa, chest radiography, and hand X-rays showed no significant abnormalities. However, radiographs of the lower legs demonstrated characteristic periosteal thickening along the cortical shafts (Fig. 2).

### 2.3. Genetic testing and diagnosis

Given the classic clinical and radiological findings, a diagnosis of primary HOA, namely PDP, was made. Due to the known genetic etiology involving mutations in the HPGD or SLCO2A1 genes, confirmatory genetic testing via Sanger sequencing or a targeted next-generation sequencing panel was strongly recommended for definitive diagnosis and familial counseling. After detailed discussion, the patient and his family declined genetic testing due to personal preference and financial considerations.

### 2.4. Treatment

A symptomatic management plan was initiated. The patient was prescribed oral isotretinoin (20 mg once daily) to address cutaneous manifestations (sebaceous hyperplasia and pachyderma). For joint pain and swelling, diclofenac (50 mg twice daily), a non-steroidal anti-inflammatory drug, was administered.

### 2.5. Follow-up

At the three-month follow-up, the patient reported significant subjective improvement, including reduced facial oiliness and markedly decreased knee pain and swelling, leading to improved mobility. Objective assessment showed softening of the cutaneous folds. After 5 years of ongoing follow-up, his disease entered a stable plateau phase with no further progression of skin thickening or arthropathy. No systemic complications, such as myelofibrosis, developed during this period.

## 3. Discussion

PDP represents the primary manifestation of HOA, also known as Touraine-Solente-Gole syndrome, a rare hereditary disorder with suggested autosomal dominant and autosomal recessive inheritance patterns. Genomic studies have pinpointed mutations in 2 key genes within the PGE2 metabolism pathway: HPGD, responsible for encoding HPGD, the primary enzyme involved in PGE2 degradation, and SLCO2A1, responsible for encoding a transporter protein pivotal in PGE2 uptake and clearance. Mutations in either gene result in sustained high levels of PGE2, leading to diverse clinical and biochemical presentations. Notably, individuals with mutations in HPGD may exhibit distinct features compared to those with mutations in SLCO2A1.^[5]^ Based on the research conducted by Seta et al, individuals with SLCO2A1 mutations tend to develop a more severe form of the disease, often exhibiting later onset and a higher likelihood of presenting with cutis verticis gyrata and joint involvement compared to those with HPGD mutations.^[6]^ One study indicates that mutations within the SLCO2A1 gene frequently contribute to the pathogenesis of PDP, particularly among Asian-Indian cohorts.^[7]^

The hallmark clinical features of PDP include pachyderma, digital clubbing, and periostosis. Unlike secondary HOA, PDP is characterized by widespread and pronounced cutaneous overgrowth, often leading to facial and scalp alterations such as leonine facies and cutis verticis gyrata, respectively. Glandular hypertrophy manifests as seborrhea, ptosis, acne, and hyperhidrosis.^[1]^ Histological examination of pachydermic lesions typically reveals mucin deposition, dermal edema, and elastic fiber degeneration in the early stages, progressing to fibrosis and sebaceous gland hyperplasia in advanced cases.^[8]^ Digital clubbing, a consequence of edema and soft tissue enlargement, can result in a characteristic drumstick appearance of the nails, while lower limb soft tissue swelling may resemble elephantiasis.^[1]^ Periostosis, visualized radiologically, is a defining feature, particularly evident along the shafts of tubular bones. Patients may present with arthritis or arthralgia, with synovial effusion potentially observed in the knees.^[4]^

Differential diagnosis is crucial to distinguish PDP from secondary HOA, which often accompanies underlying pulmonary, cardiac, hepatic, gastrointestinal, or mediastinal diseases and malignancies.^[1]^ Additionally, conditions such as thyroid acropachy, acromegaly, and POEMS syndrome (polyneuropathy, organomegaly, endocrinopathy, monoclonal gammopathy and skin changes) share clinical and radiological similarities with PDP.^[1,4]^ The diagnosis of PDP remains clinical, supported by radiological findings.^[9]^ The normal ALP and BMD in our patient help differentiate PDP from metabolic bone diseases, although osteoporosis has been documented in advanced PDP cases, warranting potential BMD monitoring.^[10]^

While radiographs are fundamental, advanced imaging techniques offer deeper insights. Techniques like diffusion-weighted whole-body imaging with background signal suppression and positron emission tomography have shown utility in assessing the metabolic activity and extent of disease in HOA, potentially aiding in monitoring treatment response in complex cases.^[11,12]^ Technetium-99m methylene diphosphonate bone scintigraphy is highly sensitive for detecting early periostitis, showing symmetrical, diffuse tracer uptake along the cortical margins of long bones, including the epiphyses, which helps differentiate it from secondary HOA and thyroid acropachy.^[13,14]^

Therapeutically, non-steroidal anti-inflammatory drugs are commonly employed in PDP management,^[15]^ although their impact on structural hypertrophy progression requires further investigation.^[1]^ One study indicates that colchicine can alleviate joint symptoms, folliculitis, and pachyderma, while isotretinoin improves pachyderma and cutis verticis gyrata.^[16]^ Similarly, another article suggests that the combination of etoricoxib and aescin significantly reduces joint pain and inflammation without compromising liver and renal function during a 1-year follow-up period.^[17]^ A recent study has shown that patients treated with etoricoxib exhibited a positive response after 7 months, with noticeable improvements in clinical symptoms such as profuse sweating, thickening of the skin on the forehead, and easy fatigability.^[18]^ For patients with significant bone turnover, intravenous bisphosphonates (e.g., zoledronic acid) have been used successfully, leading to a marked decrease in bone-specific ALP levels and potentially mitigating bony overgrowth. For refractory cutaneous manifestations, emerging treatments such as phosphodiesterase-4 (PDE-4) inhibitors, Janus kinase (JAK) inhibitors, and biologic agents like dupilumab have been explored with some benefit.^[19]^ Botulinum toxin type A injection represents a straightforward yet temporary cosmetic intervention for improving pachydermia.^[20]^ For severe joint deformity or cutis verticis gyrata, surgical correction can be considered.^[21]^ Generally, PDP exhibits a self-limiting course, with cutaneous and skeletal changes progressing over several years before stabilizing. However, regular monitoring is advisable to detect potential complications such as myelofibrosis.^[4]^ Furthermore, in light of reports describing vertebral involvement and severe myelopathy, long-term follow-up should include vigilance for neurological symptoms related to spinal canal or foraminal stenosis, which may require surgical intervention.^[19]^

## Acknowledgments

We would like to thank the Department of Radiology of our hospital for their assistance in imaging interpretation.

## Author contributions

**Conceptualization:** Min Li, Zhou Zhou.

**Data curation:** Xiaohan Hu, Min Li, Zhou Zhou.

**Formal analysis:** Zhou Zhou.

**Investigation:** Xiaohan Hu.

**Methodology:** Zhou Zhou.

**Project administration:** Min Li.

**Resources:** Zhou Zhou.

**Supervision:** Min Li.

**Validation:** Min Li.

**Writing – original draft:** Xiaohan Hu, Zhou Zhou.

**Writing – review & editing:** Min Li.
